# Clinical Reports

**Published:** 1880-04

**Authors:** Donald Maclean

**Affiliations:** Professor of Surgery in the University of Michigan


### CLINICAL REPORTS.

### *SURGICAL CLINIC AT UNIVERSITY OF MICHIGAN.*

BY DONALD MACLEAN, M. D.,
Professor of Surgery in the University of Michigan.

### EXCISION OF ONE-HALF OF THE LOWER JAW.

You will remember that this man was operated upon for a malignant growth upon the right side of the lower jaw one week ago to-day. The tissue was laid back and the inferior maxillary bone was divided and the diseased portion removed. The wound has healed rapidly and there has been sloughing at only one point. The parotid gland was wounded and on this account the patient has lost much saliva. At a distance of three or four feet the line of incision cannot now be detected. That side of the face now droops and appears to be swollen. This is due to want of support, and soon the place of the excised bone will be filled with hard fibrous tissue upon which false teeth can be fitted and this support will obviate the present slight deformity. You will perceive that the patient can close and wink his eye and smile. This shows that the facial nerve was not wounded, and it was avoided by taking care not to extend the incision back to the ear.

PAROTID TUMOR.—This little boy has a large adenoid tumor situated just under the parotid gland. It is rapidly increasing in size, and as there is scarcely any limit to the growth of these tumors, excision is the only means of relief. I once removed one which weighed fifteen pounds and which hung down over the man's chest. It must be remembered that these tumors are called "parotid" simply because they are in the region of that gland, and the name does not necessarily imply that the gland is involved. One or two surgeons claim to have successfully removed the parotid gland, but the probability is that they have been mistaken and have removed only tumors lying over the true gland. Removal of the parotid gland would involve so many important structures that it must yet be regarded as a very questionable operation to say the least.

The little boy now being under the influence of chloroform, I make an incision and easily turn out the tumor, taking care not to wound the external jugular vein.

*The Physician and Surgeon.*

